# Beyond symptoms: wearable digital biomarkers for subclinical detection of chemotherapy-induced peripheral neuropathy

**DOI:** 10.3389/fonc.2026.1959858

**Published:** 2026-09-24

**Authors:** Di Xu

**Affiliations:** Department of Cardiac Surgery, The First Affiliated Hospital of Xi'an Jiaotong University, Xi’an, Shaanxi, China

**Keywords:** chemotherapy-induced peripheral neuropathy, digital biomarkers, electrochemical skin conductance, gait analysis, inertial wearable monitoring, patient-reported outcomes, subclinical detection, wearable sensors

## Abstract

**Background:**

Chemotherapy-induced peripheral neuropathy (CIPN) is the most prevalent dose-limiting toxicity of taxane-, platinum-, and proteasome inhibitor-based regimens, yet clinical management has a structural flaw: assessment depends on CTCAE clinician grading that registers symptoms only after axonal degeneration has reached an irreversible stage.

**Main body:**

Quantitative sensory testing studies demonstrate that vibration perception, thermal detection, and sudomotor function deteriorate before patients report numbness or pain, suggesting a subclinical window of one to two chemotherapy cycles. Whether detection within this window can prevent permanent nerve injury remains biologically plausible but prospectively unestablished. Wearable sensors—inertial measurement units for gait, plantar pressure insoles, electrochemical skin conductance devices, and vibration sensors—have demonstrated proof-of-concept capacity to capture subclinical CIPN signals, but evidence remains largely cross-sectional with limited longitudinal validation. This Perspective distinguishes the conceptual framework for subclinical CIPN surveillance from the currently available clinical validation evidence. I systematically categorize four sensing modalities by neuropathological substrate, provide a cross-modality readiness comparison, and identify translational barriers including absent alert thresholds and confounding factors such as age, diabetes, anemia, and fatigue. I propose a phased prospective validation framework; specific monitoring recommendations should be regarded as hypotheses for testing rather than current clinical guidance.

**Conclusion:**

Wearable sensors should not replace patient-reported outcomes but may add an objective neurotoxicity monitoring layer whose capacity to improve clinical outcomes requires prospective validation.

## 1 Introduction

Chemotherapy-induced peripheral neuropathy (CIPN) affects 30–70% of patients receiving taxanes, platinum compounds, vinca alkaloids, or proteasome inhibitors and represents the single most common dose-limiting toxicity in medical oncology (1–3). The clinical burden extends beyond sensory discomfort: CIPN increases fall risk by approximately 1.5- to 2-fold, impairs gait and balance, degrades quality of life, and frequently necessitates dose reduction or early discontinuation of potentially curative chemotherapy (4–6). Among breast cancer survivors, long-term CIPN prevalence exceeds 40% at five years post-treatment, and the condition is increasingly recognized as a chronic cancer pain syndrome rather than a transient treatment toxicity (7).

The standard tool for CIPN grading in oncology practice is the National Cancer Institute CTCAE, a clinician-rated 5-point scale (8). Despite its universal adoption, CTCAE has well-documented limitations: inter-rater reliability is modest, agreement between clinician-rated and patient-reported severity is poor, and—most critically for the present argument—the scale registers symptoms, not pathology (9, 10). A patient receives a CTCAE grade ≥2 only when functional limitation from sensory symptoms becomes evident, but by that point intraepidermal nerve fiber density has already declined and large myelinated fibers show axonal degeneration that has passed from reversible metabolic dysfunction to structural injury (11, 12). Moreover, CTCAE grading is performed at infrequent, discrete clinic visits and provides no serial objective measurement, leaving subclinical neurotoxicity entirely unmonitored in the intervals between assessments—a gap that motivates a continuous, sensor-based monitoring approach.

The consequence is a temporal disconnect between when nerve injury begins and when current tools detect it. Quantitative sensory testing (QST) studies have demonstrated that vibration perception thresholds rise, cold detection thresholds deteriorate, and sudomotor function declines before patients endorse sensory symptoms on validated patient-reported outcome (PRO) measures (13–15). In a longitudinal cohort of oxaliplatin-treated patients, vibration thresholds increased by approximately 30% from baseline before CTCAE grade changed (13), and foot electrochemical skin conductance (ESC) declined by approximately 20% after two chemotherapy cycles, preceding patient-reported symptom onset (14). These subclinical changes are not subtle, and they create a clinical window—potentially one to two chemotherapy cycles wide—during which dose adjustment or neuroprotective intervention could prevent permanent nerve injury. That subclinical detection window is currently unexploited because no objective, scalable monitoring tool has been integrated into routine oncology practice.

Several excellent reviews have addressed CIPN pathophysiology (2), pharmacological and non-pharmacological treatment strategies (3), and the psychometric properties of PRO instruments for CIPN assessment (16). Parallel reviews in the engineering literature have surveyed wearable sensor platforms for neurological gait analysis (17–19) and digital biomarker development frameworks (20–22), and wearable monitoring systems across physiological domains (23, 24). However, these two literatures have remained largely separate: clinical reviews do not systematically evaluate sensor readiness for subclinical CIPN detection, and engineering reviews do not anchor sensor selection to the specific neuropathological domains (large-fiber, small-fiber, and functional compensation) that define CIPN progression. This Perspective bridges that gap by (a) categorizing four wearable sensing modalities—IMU-based gait analysis, plantar pressure insoles, ESC, and wearable quantitative vibration sensors—according to their target neuropathological substrate rather than their hardware platform; (b) providing a cross-modality comparison of detection advantage, limitation, and clinical readiness; and (c) identifying the translational barriers that must be resolved before sensor-derived signals can inform chemotherapy dosing decisions. This Perspective focuses on subclinical CIPN detection rather than CIPN treatment; the clinically relevant interventions that early detection enables—dose modification, treatment rotation, neuromuscular training (6), and cryotherapy (25)—are addressed elsewhere. The clinical levers that detection is intended to inform exert their effect on CIPN incidence and progression: dose reduction lowers cumulative neurotoxic exposure and attenuates neuropathy but may compromise oncological efficacy, whereas cryotherapy reduces taxane-induced neuropathy in a limb-distal pattern. My concern is the upstream question: can I detect CIPN before the patient feels it, and if so, what stands between current sensor technology and that clinical reality?

Literature search and selection strategy. This Perspective synthesizes literature identified through a structured but narrative (non-systematic) search, which is the appropriate approach for this article type rather than a PRISMA-conformant systematic review. I searched PubMed and Scopus for records published between January 2010 and July 2026 using combinations of the terms ‘chemotherapy-induced peripheral neuropathy’, ‘peripheral neuropathy’, ‘wearable sensors’, ‘inertial measurement unit’, ‘gait analysis’, ‘plantar pressure’, ‘electrochemical skin conductance’, ‘vibration perception threshold’, and ‘digital biomarker’. Records were screened by title and abstract for relevance to subclinical detection, sensor-based assessment, or digital biomarker development in CIPN or a closely related length-dependent neuropathy (e.g., diabetic neuropathy), and were screened backwards via the reference lists of retrieved reviews. I prioritized (i) studies reporting quantitative sensor or quantitative sensory testing (QST) measures against a clinical or neurophysiological reference, (ii) systematic reviews and meta-analyses, and (iii) regulatory or methodological frameworks for digital biomarker qualification. The four sensor modalities treated in detail—IMU-based gait analysis, plantar pressure insoles, electrochemical skin conductance, and wearable vibration/tactile sensing—were selected because each interrogates a distinct neuropathological domain (large-fiber, small-fiber, or functional compensation) and each has demonstrated at least proof-of-concept capacity in CIPN or an analogous neuropathy. Complementary but non-wearable methods—corneal confocal microscopy, skin punch biopsy for intraepidermal nerve fiber density, and nerve conduction studies—are acknowledged as reference standards and are discussed where relevant, but lie outside the wearable scope of this framework.

## 2 The subclinical window: nerve injury precedes symptom report

CIPN follows a length-dependent, dying-back pattern in which the longest sensory axons in the distal extremities are affected first. The earliest pathological changes—intraepidermal nerve fiber terminal swelling, mitochondrial dysfunction in dorsal root ganglion neurons, and impaired axonal transport—precede conscious sensory awareness (11, 26). As degeneration progresses proximally, large myelinated Aβ fibers mediating vibration and proprioception and small unmyelinated C fibers mediating thermal and autonomic function become sequentially involved (12). By the time a patient notices numbness in the toes, the distal axonopathy is already structurally established.

QST provides quantitative confirmation of this temporal sequence. A landmark study by Krøigård et al. in 85 oxaliplatin-treated patients demonstrated that vibration perception threshold at the hallux increased by a mean of 2.3/8 units (approximately 30% from baseline) before any CTCAE grade change (13). Cold and warm detection thresholds showed similar pre-clinical drift. In a separate cohort, foot ESC measured by Sudoscan declined significantly after the second cycle of oxaliplatin, again preceding patient-reported symptom onset, with a decline of approximately 20% from baseline correlating with clinically significant CIPN at six months (14). These findings are consistent across chemotherapy classes: paclitaxel produces the same pattern of subclinical small-fiber dysfunction detectable by quantitative sudomotor axon reflex testing before symptom report (27). A systematic review and meta-analysis across 31 studies (n = 1,635) confirmed moderate-to-large increases in vibration perception threshold after neurotoxic chemotherapy in both the hands (standardized mean difference 0.75) and the feet (standardized mean difference 0.69), demonstrating that subclinical large-fiber dysfunction is detectable at both distal sites but is slightly more pronounced in the feet—consistent with the length-dependent gradient (28). This Perspective therefore frames subclinical detection primarily around the feet and lower limbs, which are affected earliest and most severely, while noting that upper-limb vibration measures are also sensitive and may be preferable in specific settings (e.g., where the hands are the site of functional need or where a portable hand-held device is more practical).

The clinical implication is biologically plausible but has not yet been prospectively established. If sensor technology can reliably detect a 30% change in vibration threshold or a 15–20% decline in foot ESC, the oncologist may gain a decision window during which dose modification, treatment rotation, or a neuroprotective intervention such as neuromuscular training (6) could reduce the risk of irreversible nerve injury. I emphasize that this causal chain—from sensor-detected change, to modified treatment, to preserved nerve function—remains a hypothesis requiring prospective validation rather than an established clinical strategy.

## 3 Wearable sensors for digital phenotyping of CIPN

Rather than cataloguing devices by hardware platform—a format that obscures clinical utility—I organize the four wearable modalities relevant to subclinical CIPN detection by the neuropathological domain they interrogate: large-fiber function (IMU-based gait analysis and wearable vibration sensing), small-fiber function (electrochemical skin conductance), and functional compensation (plantar pressure distribution). **Table 1** provides a cross-modality comparison of core signal, detection advantage, limitation, clinical readiness, and optimal monitoring scenario.

**Table 1:** Cross-modality comparison of wearable sensors for subclinical CIPN detection.

Modality	Core signal	Detection advantage	Key limitation	Clinical readiness	Optimal monitoring scene
IMU-based Gait/Balance (Large-fiber proprioception)	Trunk acceleration, stride time variability, double-support phase, postural sway area	Captures functional consequence of sensory loss; validated against fall risk (OR ~1.7 per SD increase in sway) (4, 29)	Non-specific: fatigue, cachexia, anemia independently affect gait; requires standardized walk protocol	Moderate: cross-sectional studies (n > 200), limited longitudinal within-patient data	Weekly 2-min walk test; clinic + home; curative-intent chemotherapy
Plantar Pressure Insoles (Functional compensation)	Center-of-pressure trajectory, foot loading redistribution (toe→midfoot shift), pressure-time integral	Directly measures consequence of plantar sensory loss; ecologically valid during daily activity (30)	Consumer-grade insole accuracy vs. force plate not fully validated in CIPN populations; normative data absent	Low: feasibility studies only (n < 50); no dedicated CIPN longitudinal validation	Clinic-based 10-m walk; complement to IMU; research setting predominantly
Electrochemical Skin Conductance (Small-fiber sudomotor)	Chloride ion flux at palms/soles under low-voltage DC; proxy for sweat gland C-fiber innervation	Rapid (<3 min), objective, no patient effort; foot ESC decline ~20% after 2 cycles precedes symptoms (14, 31)	Current devices are tabletop (not wearable continuous); influenced by ambient temperature, hydration, skin condition	Moderate-High: replicated across oxaliplatin and paclitaxel cohorts; sensitivity ~78%, specificity ~82% for grade ≥2 CIPN (31)	Clinic visit at each chemotherapy cycle; best combined with IMU for dual fiber-type coverage
Wearable Vibration/Tactile (Large-fiber Aβ threshold)	Vibration perception threshold (multi-frequency), tactile spatial acuity mapping (“glove-and-stocking”)	Closest to gold-standard QST; established subclinical sensitivity (30% threshold increase pre-CTCAE) (13)	No validated wearable form factor for CIPN; current prototypes adapted from diabetic neuropathy; test-retest data absent	Low: proof-of-concept only; prototype smart textiles lack CIPN-specific validation	Clinic QST supplement; future home-based with validated smart-sock/-glove platforms

CIPN, chemotherapy-induced peripheral neuropathy; CTCAE, Common Terminology Criteria for Adverse Events; ESC, electrochemical skin conductance; IMU, inertial measurement unit; QST, quantitative sensory testing; SD, standard deviation; OR, odds ratio.

I emphasize that the evidence summarized in this section and in **Table 1** derives predominantly from cross-sectional and proof-of-concept studies. The framework proposed here should therefore be regarded as a conceptual organizing structure for future validation rather than an evidence-based clinical protocol. Longitudinal validation in prospective cohorts, as outlined in Section 6, remains the critical outstanding step.

### 3.1 Large-fiber dysfunction: IMU-based gait analysis and wearable vibration sensing

Large myelinated Aβ fibers mediating proprioception and vibration sensation are consistently affected in CIPN, and their dysfunction manifests in two measurable domains: gait deterioration and elevated sensory thresholds. IMU-based gait analysis captures the functional consequence of proprioceptive loss. Winters-Stone et al. demonstrated that breast cancer survivors with CIPN showed significantly increased mediolateral trunk acceleration, reduced gait speed, and prolonged double-support phase compared to cancer survivors without CIPN, with effect sizes sufficient to discriminate groups using a single waist-worn IMU during a 30-second walk test (32). A recent digital balance and gait assessment platform using smartphone-based inertial sensing showed that CIPN severity correlated with increased postural sway area (r = 0.51, p < 0.001) and decreased turn velocity during the Timed Up and Go test (29). Importantly, each standard deviation increase in mediolateral sway was associated with a 1.7-fold increase in fall risk over 12 months in a cohort of 234 cancer survivors (4). Even patients classified as CTCAE grade 1 (minimal or no functional limitation) exhibit decreased stride length and increased stride time variability compared to chemotherapy-naive controls (33), indicating that gait parameters capture the functional consequences of sensory loss before the patient consciously perceives the deficit. A standardized 2-minute walk test with a waist-worn IMU minimizes motion artifact and offers a pragmatic balance of signal quality and patient burden. Monfort and colleagues further showed that gait and balance impairments emerge during taxane-based chemotherapy in early-stage breast cancer patients, consistent with subclinical functional decline preceding symptomatic CIPN (34).

Wearable quantitative vibration sensing is earlier in its development trajectory. Traditional QST instruments require trained operators and clinic visits, but miniaturized vibrating actuators integrated into socks or handheld devices with Bluetooth connectivity have demonstrated test-retest reliability comparable to laboratory QST in diabetic neuropathy (35), though dedicated CIPN validation is absent. Smart textile gloves and socks with embedded pressure sensor arrays can map the spatial distribution of tactile sensitivity loss—the classic glove-and-stocking pattern of CIPN—but remain at the feasibility stage (36). Critically, the measurement procedures themselves require validation. Dujmović and colleagues showed that the practical application of QST in CIPN is sensitive to methodological detail—stimulus parameters, test duration, and site—underscoring that sensor-specific acquisition settings must be harmonized and validated before clinical deployment (37).

### 3.2 Small-fiber dysfunction: electrochemical skin conductance

Small unmyelinated C fibers innervating sweat glands are among the earliest neural structures affected by neurotoxic chemotherapy, and their dysfunction is invisible to conventional large-fiber assessments such as nerve conduction studies (14). ESC applies a low-voltage direct current to the palms and soles and measures chloride ion flux generated by activated sweat glands, providing a quantitative readout of sudomotor nerve integrity in under three minutes with no patient effort. In patients receiving oxaliplatin, foot ESC declined by approximately 20% from baseline after two cycles, with the decline correlating with clinically significant CIPN at six months (14). In paclitaxel-treated patients, ESC asymmetry between feet predicted progression to CTCAE grade ≥2 CIPN with 78% sensitivity and 82% specificity (31). The strength of ESC lies in its objectivity and rapid acquisition, but current devices are tabletop instruments requiring clinic visits. Emerging flexible epidermal electrode arrays capable of continuous electrodermal activity monitoring during daily life may close this form-factor gap, enabling longitudinal sudomotor surveillance without repeated clinic attendance.

### 3.3 Functional compensation: plantar pressure distribution

Loss of plantar protective sensation alters foot loading patterns in ways that precede conscious awareness and contribute to the elevated fall risk observed in CIPN patients. Instrumented walkway and in-shoe pressure measurement studies have documented a characteristic shift of loading from the toes and metatarsal heads toward the midfoot and heel, with increased center-of-pressure trajectory variability during stance phase (16, 30). Dynamic mode decomposition of plantar pressure data from women with cancer revealed that the temporal evolution of pressure patterns diverged between patients with and without CIPN, suggesting that the signal contains information beyond what static snapshot metrics capture (30). Textile-based pressure insoles with wireless data transmission are increasingly available and have been validated against force plate gold standards for gait analysis (38). Their key advantage for CIPN monitoring is ecological validity: continuous data collection during daily activity rather than a single clinic visit snapshot. However, normative data in CIPN populations and longitudinal within-patient validation are both absent.

## 4 From sensor data to clinical decision: the missing translation layer

The technologies described above generate continuous data streams. The clinical challenge is data interpretation. Three translational gaps require resolution before sensor-derived signals can inform chemotherapy management.

First, no validated alert threshold exists for any sensor modality in CIPN. What magnitude of IMU-derived stride time variability increase should trigger oncologist notification? What percentage decline in foot ESC warrants consideration of dose reduction? The experience of diabetic neuropathy provides an instructive analogue: the vibration perception threshold of >25 V on the Biothesiometer, indicating high risk of foot ulceration, required decades of prospective cohort data to achieve consensus acceptance (35). CIPN monitoring must undergo a similar process of linking sensor outputs to clinically meaningful endpoints—functional decline, falls, chemotherapy dose modification, or persistent neuropathy at 12 months. Beyond the absence of validated thresholds, the procedures themselves—vibration stimulus frequency and amplitude, test duration, sensor placement, foot zone of interest, and ambient conditions—must be standardized and validated, because small methodological differences materially affect QST- and sensor-derived measures, as shown in recent CIPN-specific methodological work (37).

Consistent with the absence of validated thresholds, I emphasize that any specific monitoring frequency or sensor-triggered dose adjustment described in this manuscript is proposed as a hypothesis for future testing, not as a current clinical recommendation.

The threshold development pathway should proceed from analytical validation (sensor accuracy against force plate or NCS reference standards), through clinical validation (sensitivity and specificity for predicting CTCAE grade ≥2 CIPN at 6–12 months), to clinical utility (demonstration that sensor-guided dose modification reduces persistent CIPN without compromising oncological outcomes). This three-tier framework, adapted from the FDA’s digital endpoint qualification pathway (39), provides a structured alternative to the current *ad hoc* approach.

Second, the relationship between sensor-derived measures and PROs requires careful calibration. The goal of sensor-based monitoring is not to replace patient report—neuropathic symptoms are intrinsically subjective and PRO-CTCAE and EORTC QLQ-CIPN20 capture the lived experience with well-established validity (9, 40)—but to complement it with information the patient cannot provide. Sensor data should be positioned as an orthogonal dimension: what the body does versus what the patient feels. Discordance between the two (e.g., declining gait symmetry without increased pain report) may be the most clinically informative signal of all, identifying patients whose functional compensation masks progressive nerve injury. Emerging evidence suggests that a multi-modal approach—for instance, combining IMU gait metrics with foot ESC to simultaneously assess large-fiber and small-fiber integrity—could improve specificity over single-modality monitoring by reducing false positives from non-neuropathic causes of gait change such as fatigue or anemia.

Third, the remote monitoring model is technically feasible but operationally untested at scale. The NeuroDetect iOS application, which administers remote vibration and balance tests between chemotherapy cycles, demonstrated 84% adherence over 12 weeks in a 42-patient observational cohort with patient satisfaction above 4.5/5 (15). Whether remote monitoring data can influence real-time chemotherapy dosing decisions requires prospective randomized evidence that does not yet exist. **Figure 1** maps this translation pathway from signal domains through data integration to clinical decision, anchored by a three-level validation framework.

**Figure 1:**
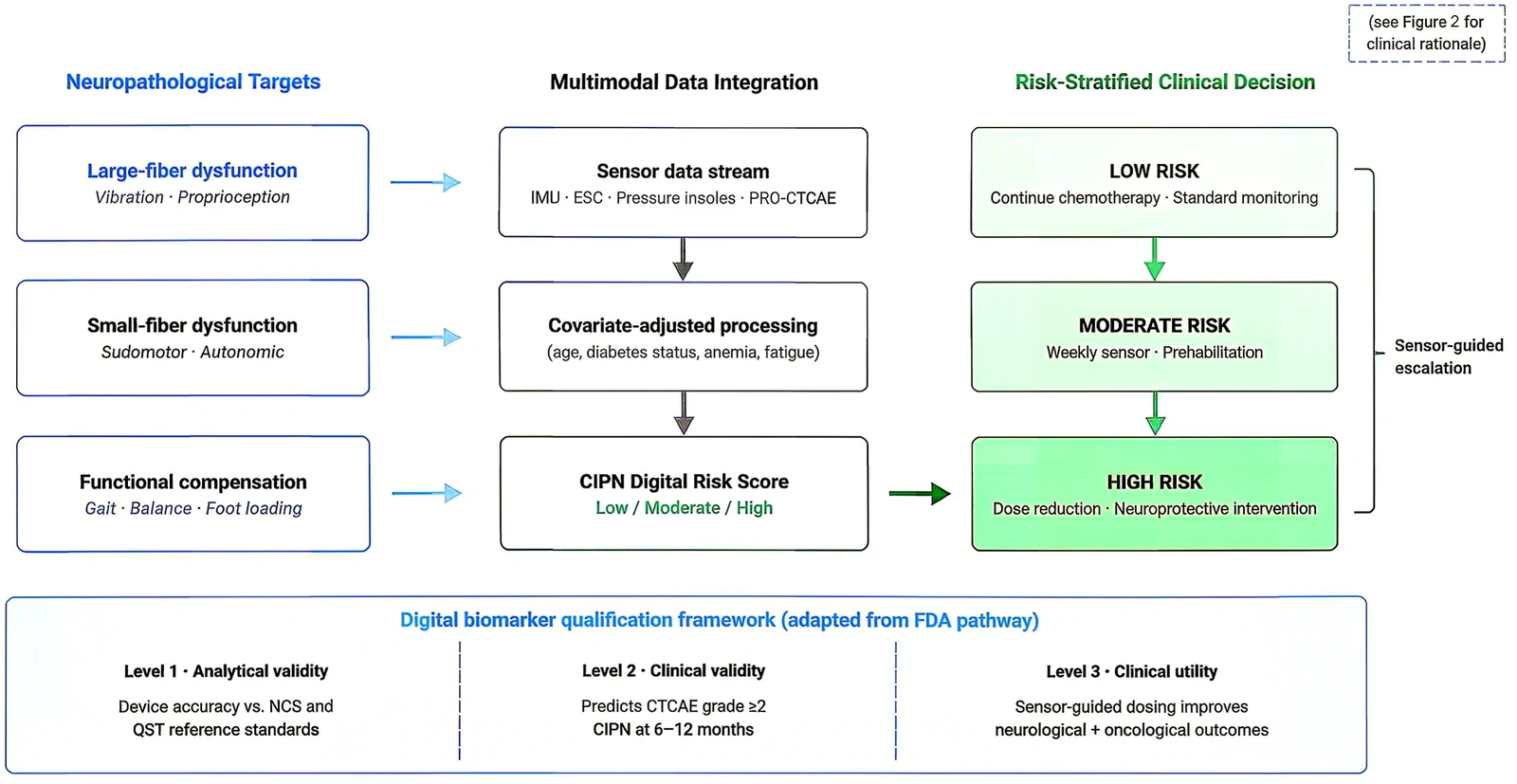
From sensor signals to clinical decisions: a digital biomarker framework for subclinical CIPN surveillance. CIPN, chemotherapy-induced peripheral neuropathy; CTCAE, Common Terminology Criteria for Adverse Events; ESC, electrochemical skin conductance; IMU, inertial measurement unit; NCS, nerve conduction studies; PRO, patient-reported outcome; QST, quantitative sensory testing.

## 5 Unresolved challenges

### 5.1 Clinical population heterogeneity

The clinical value proposition of wearable CIPN monitoring weakens as prognosis shortens, and the oncology population spans a wide range of treatment intents. For patients receiving curative-intent chemotherapy (e.g., adjuvant oxaliplatin for stage III colorectal cancer or adjuvant paclitaxel for HER2-positive breast cancer), the benefit of early CIPN detection is clearest: preserving the ability to deliver full-dose chemotherapy by preventing dose-limiting neurotoxicity has direct survival relevance. For patients receiving palliative chemotherapy where dose intensity has minimal survival impact, the monitoring emphasis should shift toward fall prevention and quality-of-life preservation. A stratified monitoring strategy—curative patients receive weekly sensor assessment with dose-modification alerts; palliative patients receive monthly assessment with fall-risk alerts—would match monitoring intensity to clinical benefit.

Beyond the prognosis-dependent monitoring question, the oncology population carries a high burden of conditions that independently affect the sensor modalities discussed here. Diabetes mellitus—present in 15–20% of oncology patients—produces its own length-dependent sensory neuropathy with overlapping gait, sudomotor, and vibration abnormalities. Prior (pre-chemotherapy) neuropathy from diabetes, treatment for an earlier malignancy, or other causes must be established at baseline to distinguish new-onset CIPN from pre-existing nerve damage. Advanced age alters gait speed and postural control independent of neuropathy. Anemia, fatigue, cachexia, and musculoskeletal comorbidities such as osteoarthritis further confound inertial and pressure-derived parameters. The validation framework described in Section 6 will therefore record these variables at baseline and at each follow-up, incorporate them as covariates in the analytical plan, and include stratified sensitivity analyses restricted to the subgroup without diabetic or other pre-existing neuropathy.

### 5.2 Validation gap

The evidence base for wearable CIPN detection consists primarily of cross-sectional studies comparing patients with established CIPN to healthy or chemotherapy-naive controls. Sample sizes rarely exceed 100, and longitudinal within-patient data—tracking the same individual from pre-chemotherapy baseline through subclinical changes to clinically evident neuropathy—are scarce (15, 29, 41). The most urgent need is a prospective cohort study collecting concurrent wearable sensor data, QST, nerve conduction studies, and PROs at standardized intervals. Section 6 provides a detailed study design. Until such data exist, the sensitivity and specificity of each sensor modality against neurophysiological gold standards remain approximate.

### 5.3 Patient adherence and device burden

Cancer patients during active chemotherapy experience fatigue, nausea, and frequent healthcare visits, and adding a daily sensor protocol represents a non-trivial burden. The design principle should be minimalism: a single waist-worn IMU with a once-weekly 2-minute walk test captures the highest-yield gait parameters at the lowest burden (29, 32). ESC measurement can be performed at scheduled clinic visits rather than continuously. For home-based monitoring, automated data upload and a user interface that requires minimal patient interaction are essential; the 84% adherence rate in the NeuroDetect study (15) establishes feasibility but was achieved in a motivated research cohort, and real-world adherence in unselected populations will likely be lower. Pragmatic implementation studies embedded within routine chemotherapy workflows are needed to determine sustainable adherence levels. A further consideration is the patient perspective on dose modification. Because subclinical neuropathy is asymptomatic, patients may be reluctant to accept dose reduction—which they may equate with a lower chance of cure—in the absence of objective evidence that neurotoxicity is progressing. Objective sensor-derived evidence of subclinical deterioration could support shared decision-making and improve patient acceptance of dose adjustments.

### 5.4 Regulatory and acceptance barriers

For sensor-derived CIPN biomarkers to influence clinical decisions, they must gain acceptance from oncologists, regulatory agencies, and guideline panels. The FDA’s qualification of digital mobility outcomes in Duchenne muscular dystrophy provides a procedural template (39), but CIPN-specific regulatory engagement has not occurred. Clinician acceptance requires demonstrating that sensor data add actionable information beyond what the oncologist already obtains from patient interview and CTCAE grading. A pragmatic trial randomizing oncologists to receive versus not receive a sensor-derived CIPN risk score, with the primary endpoint being the rate of chemotherapy dose modification, would directly test clinical utility. An economic argument reinforces the case for objective early detection: CIPN contributes to avoidable healthcare costs through chemotherapy dose reductions that may compromise survival, hospitalizations for falls and neuropathic pain, and long-term rehabilitation. Preserving dose intensity and preventing chronic neuropathy are therefore potentially cost-saving, a consideration that supports investment in routine sensor-based monitoring.

## 6 A phased roadmap for clinical translation

The evidence reviewed above supports the assertion that wearable sensor technology for subclinical CIPN detection has passed the proof-of-concept stage but has not demonstrated the clinical validity required for treatment decision-making. Closing this gap requires a deliberate sequence of studies rather than further platform proliferation. I propose a three-phase roadmap (Section 6.1-6.3).

### 6.1 Short-term (1–2 years): prospective single-center cohort

A prospective observational cohort (n = 150–200) enrolling patients initiating taxane- or platinum-based chemotherapy. At baseline, each chemotherapy cycle, and 3, 6, and 12 months post-treatment, participants complete: (a) a standardized wearable sensor battery (waist-worn IMU during 2-minute walk, plantar pressure insole during 10-meter walk, and foot ESC measurement at clinic visits); (b) QST (vibration, cold, and warm detection thresholds); (c) nerve conduction studies of sural and peroneal nerves; (d) PRO-CTCAE and EORTC QLQ-CIPN20; and (e) optional skin punch biopsy for intraepidermal nerve fiber density and corneal confocal microscopy in a subset. The primary analysis would establish (i) the lead time of each sensor modality relative to CTCAE grade change, (ii) the area under the receiver operating characteristic curve for predicting persistent grade ≥2 CIPN at 12 months, and (iii) sensor-derived alert thresholds with defined sensitivity and specificity. Such a dataset would be the first to establish sensor validity against concurrent neurophysiological and histological gold standards in a longitudinal CIPN cohort.

The cohort size was selected on the following grounds. Assuming a 12-month persistent CIPN (grade ≥2) incidence of 35–40% among taxane- and platinum-treated patients, consistent with the range reported in the oncology literature (1–3), and aiming to estimate sensor sensitivity of 80% with a two-sided 95% confidence interval half-width of 10 percentage points, the required number of affected patients is approximately 62 (n = z^2^p(1−p)/d^2^ = 1.96^2^ × 0.8 × 0.2/0.1^2^ ≈ 61), where n is the required number of affected patients, z = 1.96 is the standard normal deviate for a two-sided 95% confidence level, p = 0.8 is the assumed sensor sensitivity, and d = 0.1 is the half-width of the confidence interval. To obtain this number of affected patients, approximately 155–180 total enrollees are needed; allowing 10–15% attrition yields a target cohort of 180–200 participants. This size also provides ≥80% power to detect a moderate effect (Cohen’s d ≈ 0.5) in sensor-derived risk scores between patients with and without persistent CIPN at 12 months. The primary endpoint—persistent CTCAE grade ≥2 CIPN at 12 months—was selected because persistent rather than transient neuropathy is the outcome that determines long-term disability and quality of life; sensor-derived alert thresholds and chemotherapy dose modification events are secondary endpoints. The monitoring intervals (baseline, each chemotherapy cycle, and 3, 6, and 12 months post-treatment) were chosen to bracket the one-to-two-cycle subclinical window (**Figure 2**) suggested by quantitative sensory testing evidence and to capture the full temporal evolution from baseline to persistent neuropathy. To evaluate whether sensor-guided dose modification compromises oncological efficacy—a central concern for patients and clinicians alike (Section 5.3)—the study will additionally record relative dose intensity delivered and, over longer follow-up, oncological outcomes (disease-free and overall survival), with chemotherapy dose-modification events tracked as a pre-specified secondary endpoint.

**Figure 2:**
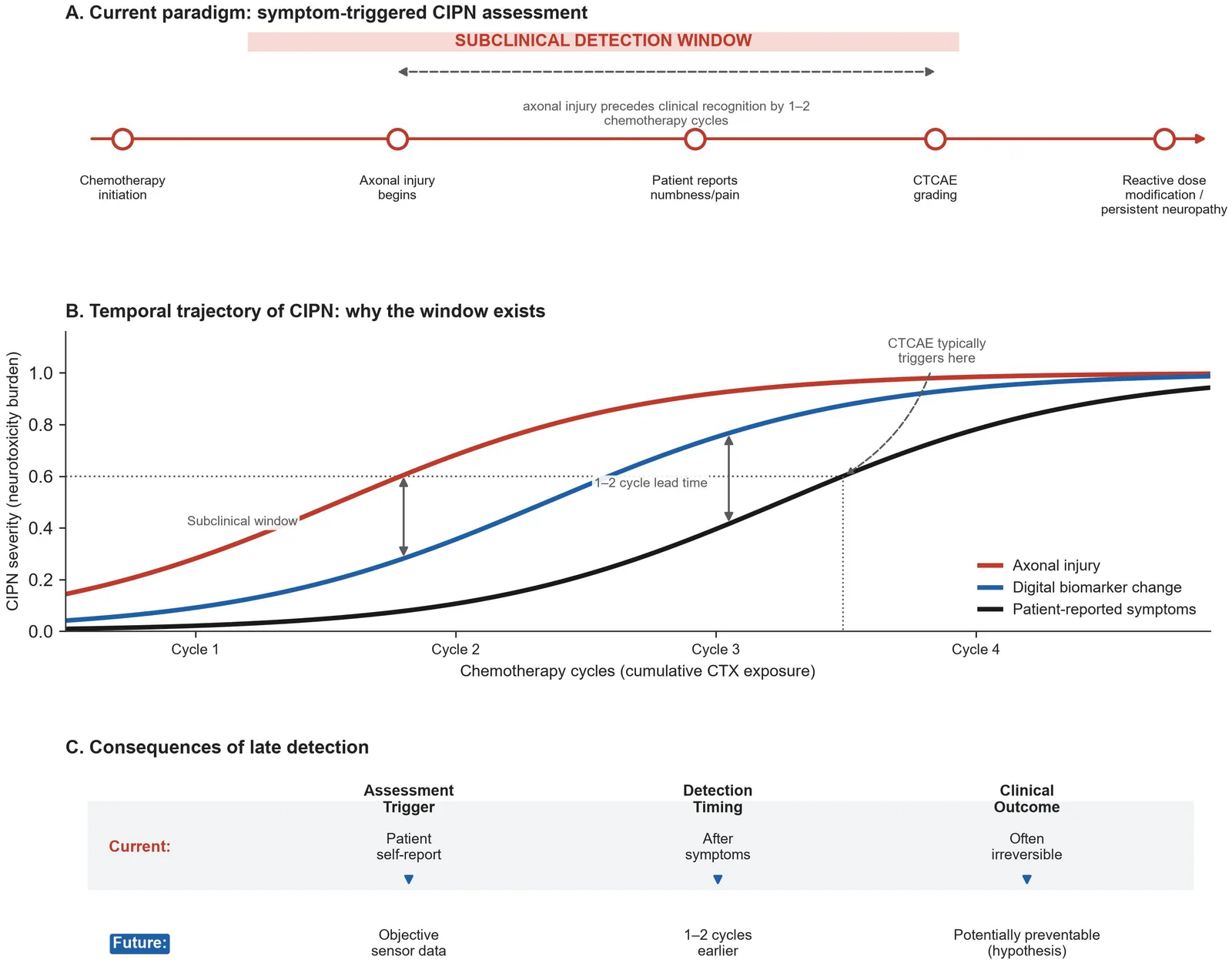
The subclinical detection window between axonal injury and clinical recognition in CIPN.

### 6.2 Mid-term (3–5 years): multicenter phase III trial integration

Oncology cooperative groups and pharmaceutical sponsors should incorporate wearable sensor endpoints into randomized phase III chemotherapy trials as secondary or exploratory objectives. This achieves two goals simultaneously: it embeds sensor data collection within the existing trial infrastructure, minimizing incremental operational burden, and it generates the large-scale, multicenter evidence required for regulatory qualification. The minimum viable sensor panel—a waist-worn IMU with weekly 2-minute walk test plus clinic-based ESC—has an acquisition cost and logistical footprint compatible with multicenter trial deployment.

### 6.3 Long-term (5+ years): CTCAE augmentation and guideline integration

If phase III data confirm that sensor-derived measures predict persistent CIPN with clinically meaningful lead time, the ultimate goal is to augment CTCAE-based neuropathy grading with objective digital adjuncts. This would parallel the evolution of cardiac toxicity monitoring, where ejection fraction measurement supplements symptomatic heart failure assessment. A pragmatic trial randomizing oncologists to CTCAE-alone versus CTCAE-plus-sensor-guided management, with persistent CIPN at 12 months as the primary endpoint, would provide the definitive evidence of clinical utility required for guideline panel consideration.

## 7 Conclusion

CTCAE-based CIPN assessment belongs to an era when clinicians had no alternative to asking the patient how they feel. Wearable IMUs can now detect gait asymmetry before the patient notices it, pressure insoles map the redistribution of foot loading as plantar sensation fades, and ESC devices quantify small-fiber dysfunction in under three minutes. These technologies capture a subclinical window during which intervention could prevent irreversible axonal injury. The subclinical detection window is currently not being exploited not because the sensor technology is immature, but because the clinical evidence linking sensor-derived signals to treatment decisions has not been generated. The roadmap proposed here—prospective validation, multicenter trial integration, and eventual CTCAE augmentation—provides a structured pathway to close that gap. The patients who would benefit most are those for whom CIPN means the difference between completing curative chemotherapy and stopping early. They deserve more than a 5-point scale designed in 1983. The sensor technology exists. The biological rationale is sound. What remains is the commitment to generate the evidence that connects what the body shows to the decisions that matter.

## Data Availability

The original contributions presented in the study are included in the article/supplementary material. Further inquiries can be directed to the corresponding author.
